# Bis(2,2′-bipyridyl-κ^2^ *N*,*N*′)(sulfato-κ^2^ *O*,*O*′)zinc(II) ethane-1,2-diol solvate

**DOI:** 10.1107/S1600536809055433

**Published:** 2010-01-09

**Authors:** Kai-Long Zhong

**Affiliations:** a Department of Applied Chemistry, Nanjing College of Chemical Technology, Nanjing 210048, People’s Republic of China

## Abstract

The title compound, [Zn(SO_4_)(C_10_H_8_N_2_)_2_]·C_2_H_6_O_2_, is a six-coordinate zinc(II) complex with a distorted octa­hedral coordination geometry. The Zn^II^ atom is bonded by two O atoms of the bidentate chelating sulfate ligand and four N atoms of the two chelating 2,2′-bipyridine ligands. The Zn—N bond distances range from 2.1287 (17) to 2.1452 (17) Å and the Zn—O bond distance is 2.1811 (15) Å. The two chelating NCCN groups subtend a dihedral angle of 81.1 (1)°. In the crystal structure, the [ZnSO_4_(C_10_H_8_N_2_)_2_] and C_2_H_6_O_2_ units are connected by inter­molecular O—H⋯O hydrogen bonding, which leads to additional stabilization of the structure.

## Related literature

For related compounds, see: Liu & Arora (1993 ▶); Harvey *et al.* (2001 ▶, 2002 ▶); Jian *et al.* (2004 ▶); Rodrigues (2004 ▶); Juric *et al.* (2006 ▶); Zhu *et al.* (2006 ▶); Yu *et al.* (2007 ▶); Zhong *et al.* (2007 ▶).
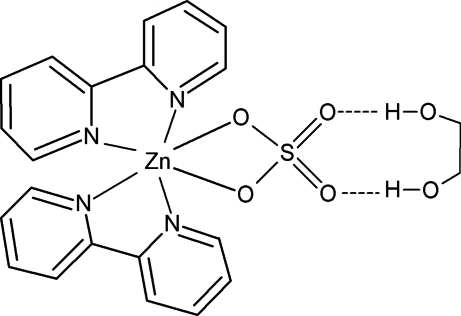


## Experimental

### 

#### Crystal data

[Zn(SO_4_)(C_10_H_8_N_2_)_2_]·C_2_H_6_O_2_
*M* *_r_* = 535.90
Monoclinic, 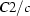

*a* = 17.017 (3) Å
*b* = 11.890 (2) Å
*c* = 12.831 (3) Å
β = 122.14 (3)°
*V* = 2198.3 (10) Å^3^
*Z* = 4
Mo *K*α radiation
μ = 1.26 mm^−1^
*T* = 223 K
0.55 × 0.45 × 0.20 mm

#### Data collection

Rigaku Mercury CCD diffractometer
Absorption correction: multi-scan (*REQAB*; Jacobson, 1998 ▶) *T*_min_ = 0.747, *T*_max_ = 1.000
6138 measured reflections
2489 independent reflections
2155 reflections with *I* > 2σ(*I*)
*R*_int_ = 0.021

#### Refinement

*R*[*F* ^2^ > 2σ(*F* ^2^)] = 0.035
*wR*(*F* ^2^) = 0.088
*S* = 1.08
2489 reflections
156 parameters
H-atom parameters constrained
Δρ_max_ = 0.66 e Å^−3^
Δρ_min_ = −0.50 e Å^−3^

### 

Data collection: *CrystalClear* (Rigaku, 2007 ▶); cell refinement: *CrystalClear*; data reduction: *CrystalClear*; program(s) used to solve structure: *SHELXS97* (Sheldrick, 2008 ▶); program(s) used to refine structure: *SHELXL97* (Sheldrick, 2008 ▶); molecular graphics: *XP* in *SHELXTL* (Sheldrick, 2008 ▶); software used to prepare material for publication: *SHELXTL*.

## Supplementary Material

Crystal structure: contains datablocks global, I. DOI: 10.1107/S1600536809055433/bq2188sup1.cif

Structure factors: contains datablocks I. DOI: 10.1107/S1600536809055433/bq2188Isup2.hkl

Additional supplementary materials:  crystallographic information; 3D view; checkCIF report

## Figures and Tables

**Table:** 

Zn1—N2	2.1287 (17)
Zn1—N1	2.1452 (17)
Zn1—O1	2.1811 (15)
S1—O2	1.4683 (15)
S1—O1	1.4915 (15)

**Table:** 

N2—Zn1—N1	76.61 (7)
O1^i^—Zn1—O1	65.64 (8)

Symmetry code: (i) 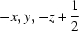.

**Table 2:** Hydrogen-bond geometry (Å, °)

*D*—H⋯*A*	*D*—H	H⋯*A*	*D*⋯*A*	*D*—H⋯*A*
O3—H3⋯O2	0.82	1.97	2.746 (2)	158

**Fig. 1.**
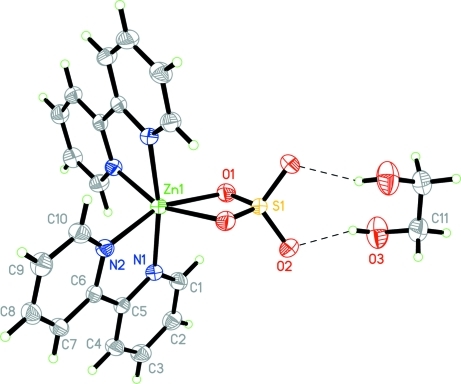
The molecular structure of (I) showing the atom-numbering scheme and displacement ellipsoids drawn at the 50% probability level. The dashed lines represent O—H···O interactions. Unlabeled atoms are related to the labeled atoms by the symmetry operator(-x, y, -z + 1/2).

## supplementary crystallographic information

### Comment

2,2'-bipyridyl(2,2'-bipy) is a well known neutral didentate ligand. Many metal zinc complexes with 2,2'-bipy have been previously synthesized and reported, such as bis(2,2'-bipyridyl) zinc(II) with diperchlorate (Liu & Arora, 1993), uranyl phosphate (Yu *et al.*, 2007), thiocyanate (Zhong *et al.*, 2007), oxalate (Juric *et al.*, 2006), azide (Jian *et al.*, 2004), acetate (Rodrigues, 2004). Moreover, the crystal structures of zinc complexes with monodente (Harvey *et al.*, 2003), bidentate chelating (Zhu *et al.*, 2006) and bidentate bridging sulfate (Harvey *et al.*, 2000) have been structurally characterized. Recently, we attempt to utilize polydentate and mixed ligands for the design of coordination networks. The title compound [ZnSO_4_(C_10_H_8_N_2_)_2_].C_2_H_6_O_2_, (I),  which is very similar to the zinc complex with 1,10-phenanthroline instead of 2,2'-bipyridine (Zhu *et al.*, 2006), was obtained unitentionally during an attempt to synthesize mixed-ligand complex of Zn(II) with 2,2'-bipy and melamine *via* a solvothermal reaction. The crystal structure of the title complex has not hitherto been reported.

Single crystal X-ray diffraction experiment reveal that a twofold rotation axis (symmetry code: -*x*, *y*, - *z* + 1/2) passes through the Zn, S atoms, and also through the mid-point of C—C bond of the solvent, 1,2-ethanediol molecule. The Zn (II) ions are coordinated by four N atoms from two chelating 2,2'-bipy ligands and two O atoms from a bidentate-chelating salfate ligand, in a distorted octahedral geometry (Fig. 1. and Table 1.). The Zn—N bond distances range from 2.1287 (17)Å to 2.1452 (17)Å and Zn—O bond distance is 2.1811 (15) Å. The N—Zn—N bite angle, O—Zn—O bite angle and the dihedral angle between the two chelating NCCN groups is 76.61 (7) Å, 65.64 (8) Å and 81.1 (1) Å, respectively. The metal complex and solvent components of the title compound are held together by intermolecular O3—H3···O2 hydrogen bonding, which contribute to the stabilization of the structure (Fig.1 and Table 2.).

### Experimental

The title compound was obtained unexpectedly during an attempt to synthesize mixed-ligand complex of Zn(II) with phenol and melamine *via* a solvo-thermal reaction. Colorless prism-shaped single crystals of the title compound for X-ray diffraction determination were prepared by 0.2 mmol mixing 2,2'-bipyridine, 0.1 mmol melamine, 0.1 mmol ZnSO_4_.7H_2_O, 2.0 ml 1,3-propanediol and 1.0 ml water and then placing them in a thick Pyrex tube, which was sealed and heated to 423 K for 3 days.

### Refinement

All H atoms of 2,2'-bipyridyl were positioned geometrically and allowed to ride on their attached atoms, with C—H =0.93 Å and *U*_iso_(H) = 1.2*U*_eq_(C). The H atoms of 1,2-ethanediol were locate in a difference map and then allowed to ride on their parent atoms, with C—H =0.97 Å and O—H =0.82 Å; *U*_iso_(H) = 1.2*U*_eq_(C) and 1.5*U*_eq_(O).

### Crystal data

**Table:**

[Zn(SO_4_)(C_10_H_8_N_2_)_2_]·C_2_H_6_O_2_	*F*(000) = 1104
*M**_r_* = 535.90	*D*_x_ = 1.619 Mg m^−^^3^
Monoclinic, *C*2/*c*	Mo *K*α radiation, λ = 0.71073 Å
Hall symbol: -C 2yc	Cell parameters from 5954 reflections
*a* = 17.017 (3) Å	θ = 3.4–27.5°
*b* = 11.890 (2) Å	µ = 1.26 mm^−^^1^
*c* = 12.831 (3) Å	*T* = 223 K
β = 122.14 (3)°	Prism, colorless
*V* = 2198.3 (10) Å^3^	0.55 × 0.45 × 0.20 mm
*Z* = 4	

### Data collection

**Table:**

Rigaku Mercury CCD diffractometer	2489 independent reflections
Radiation source: fine-focus sealed tube	2155 reflections with *I* > 2σ(*I*)
Graphite Monochromator	*R*_int_ = 0.021
Detector resolution: 28.5714 pixels mm^-1^	θ_max_ = 27.5°, θ_min_ = 3.4°
ω scans	*h* = −22→22
Absorption correction: multi-scan (REQAB; Jacobson, 1998)	*k* = −12→15
*T*_min_ = 0.747, *T*_max_ = 1.000	*l* = −16→11
6138 measured reflections	

### Refinement

**Table:**

Refinement on *F*^2^	Secondary atom site location: difference Fourier map
Least-squares matrix: full	Hydrogen site location: inferred from neighbouring sites
*R*[*F*^2^ > 2σ(*F*^2^)] = 0.035	H-atom parameters constrained
*wR*(*F*^2^) = 0.088	*w* = 1/[σ^2^(*F*_o_^2^) + (0.0539*P*)^2^] where *P* = (*F*_o_^2^ + 2*F*_c_^2^)/3
*S* = 1.08	(Δ/σ)_max_ < 0.001
2489 reflections	Δρ_max_ = 0.66 e Å^−^^3^
156 parameters	Δρ_min_ = −0.50 e Å^−^^3^
0 restraints	Extinction correction: *SHELXL97* (Sheldrick, 2008), Fc^*^=kFc[1+0.001xFc^2^λ^3^/sin(2θ)]^-1/4^
Primary atom site location: structure-invariant direct methods	Extinction coefficient: 0.0173 (10)

### Special details

**Table:**

Geometry. All e.s.d.'s (except the e.s.d. in the dihedral angle between two l.s. planes) are estimated using the full covariance matrix. The cell e.s.d.'s are taken into account individually in the estimation of e.s.d.'s in distances, angles and torsion angles; correlations between e.s.d.'s in cell parameters are only used when they are defined by crystal symmetry. An approximate (isotropic) treatment of cell e.s.d.'s is used for estimating e.s.d.'s involving l.s. planes.
Refinement. Refinement of *F*^2^ against ALL reflections. The weighted *R*-factor *wR* and goodness of fit *S* are based on *F*^2^, conventional *R*-factors *R* are based on *F*, with *F* set to zero for negative *F*^2^. The threshold expression of *F*^2^ > σ(*F*^2^) is used only for calculating *R*-factors(gt) *etc*. and is not relevant to the choice of reflections for refinement. *R*-factors based on *F*^2^ are statistically about twice as large as those based on *F*, and *R*- factors based on ALL data will be even larger.

### Fractional atomic coordinates and isotropic or equivalent isotropic displacement parameters (Å^2^)

**Table:**

	*x*	*y*	*z*	*U*_iso_*/*U*_eq_	
Zn1	0.0000	0.30659 (2)	0.2500	0.02342 (14)	
S1	0.0000	0.53723 (5)	0.2500	0.02518 (18)	
N2	0.09876 (11)	0.19245 (12)	0.38322 (15)	0.0241 (4)	
O1	−0.04900 (9)	0.46074 (12)	0.14153 (13)	0.0304 (3)	
O2	0.06823 (10)	0.60723 (12)	0.24320 (15)	0.0359 (4)	
C9	0.16178 (16)	0.07532 (17)	0.5611 (2)	0.0327 (5)	
H9A	0.1567	0.0452	0.6242	0.039*	
C7	0.24124 (14)	0.09388 (16)	0.45508 (19)	0.0280 (4)	
H7A	0.2911	0.0771	0.4469	0.034*	
C8	0.23603 (14)	0.04818 (17)	0.55093 (19)	0.0316 (5)	
H8A	0.2821	−0.0001	0.6074	0.038*	
C10	0.09473 (14)	0.14816 (18)	0.47609 (19)	0.0307 (4)	
H10A	0.0450	0.1670	0.4839	0.037*	
C6	0.17124 (13)	0.16470 (15)	0.37204 (18)	0.0233 (4)	
N1	0.10086 (11)	0.29012 (13)	0.19914 (16)	0.0246 (4)	
C5	0.17129 (13)	0.21769 (16)	0.26699 (18)	0.0234 (4)	
C4	0.23903 (15)	0.19633 (16)	0.2402 (2)	0.0299 (4)	
H4A	0.2869	0.1461	0.2880	0.036*	
C1	0.09742 (14)	0.34294 (18)	0.1043 (2)	0.0297 (4)	
H1A	0.0494	0.3935	0.0581	0.036*	
C2	0.16244 (16)	0.32515 (18)	0.0724 (2)	0.0326 (5)	
H2A	0.1578	0.3625	0.0057	0.039*	
C3	0.23444 (15)	0.25072 (19)	0.1417 (2)	0.0336 (5)	
H3A	0.2791	0.2374	0.1223	0.040*	
O3	0.02134 (16)	0.82127 (14)	0.14925 (19)	0.0535 (5)	
H3	0.0243	0.7602	0.1809	0.080*	
C11	0.03464 (18)	0.90768 (19)	0.2313 (3)	0.0437 (6)	
H11A	0.0316	0.9794	0.1934	0.052*	
H11B	0.0963	0.9006	0.3045	0.052*	

### Atomic displacement parameters (Å^2^)

**Table:**

	*U*^11^	*U*^22^	*U*^33^	*U*^12^	*U*^13^	*U*^23^
Zn1	0.02046 (19)	0.0218 (2)	0.0288 (2)	0.000	0.01361 (15)	0.000
S1	0.0204 (3)	0.0203 (3)	0.0342 (4)	0.000	0.0141 (3)	0.000
N2	0.0227 (8)	0.0242 (8)	0.0248 (8)	−0.0007 (6)	0.0122 (7)	−0.0012 (6)
O1	0.0258 (7)	0.0285 (7)	0.0321 (8)	0.0005 (6)	0.0120 (6)	−0.0016 (6)
O2	0.0303 (8)	0.0280 (7)	0.0520 (10)	−0.0042 (6)	0.0236 (7)	0.0030 (7)
C9	0.0364 (11)	0.0338 (11)	0.0273 (11)	0.0006 (9)	0.0166 (9)	0.0038 (9)
C7	0.0228 (9)	0.0263 (10)	0.0312 (11)	0.0022 (8)	0.0118 (8)	−0.0011 (8)
C8	0.0284 (10)	0.0290 (10)	0.0289 (11)	0.0030 (9)	0.0094 (9)	0.0030 (8)
C10	0.0296 (10)	0.0346 (11)	0.0303 (10)	0.0027 (9)	0.0176 (9)	0.0011 (9)
C6	0.0223 (9)	0.0195 (8)	0.0256 (9)	−0.0022 (7)	0.0111 (8)	−0.0042 (7)
N1	0.0219 (8)	0.0242 (8)	0.0287 (9)	−0.0002 (7)	0.0140 (7)	0.0004 (7)
C5	0.0213 (9)	0.0208 (9)	0.0261 (9)	−0.0025 (7)	0.0112 (8)	−0.0037 (7)
C4	0.0256 (10)	0.0307 (10)	0.0348 (11)	0.0036 (8)	0.0170 (9)	−0.0013 (9)
C1	0.0292 (10)	0.0297 (10)	0.0327 (11)	0.0033 (9)	0.0182 (9)	0.0056 (9)
C2	0.0358 (11)	0.0344 (11)	0.0337 (11)	−0.0045 (9)	0.0227 (10)	−0.0008 (9)
C3	0.0323 (11)	0.0372 (12)	0.0396 (12)	−0.0011 (10)	0.0247 (10)	−0.0045 (10)
O3	0.0912 (15)	0.0382 (9)	0.0511 (11)	0.0008 (9)	0.0513 (11)	0.0052 (8)
C11	0.0494 (14)	0.0302 (11)	0.0551 (16)	−0.0024 (10)	0.0301 (13)	0.0025 (10)

### Geometric parameters (Å, °)

**Table:**

Zn1—N2^i^	2.1287 (17)	C8—H8A	0.9300
Zn1—N2	2.1287 (17)	C10—H10A	0.9300
Zn1—N1	2.1452 (17)	C6—C5	1.488 (3)
Zn1—N1^i^	2.1452 (17)	N1—C1	1.343 (3)
Zn1—O1^i^	2.1811 (15)	N1—C5	1.351 (3)
Zn1—O1	2.1811 (15)	C5—C4	1.390 (3)
Zn1—S1	2.7424 (8)	C4—C3	1.385 (3)
S1—O2	1.4683 (15)	C4—H4A	0.9300
S1—O2^i^	1.4683 (14)	C1—C2	1.385 (3)
S1—O1	1.4915 (15)	C1—H1A	0.9300
S1—O1^i^	1.4915 (15)	C2—C3	1.384 (3)
N2—C10	1.338 (3)	C2—H2A	0.9300
N2—C6	1.356 (3)	C3—H3A	0.9300
C9—C8	1.376 (3)	O3—C11	1.401 (3)
C9—C10	1.385 (3)	O3—H3	0.8200
C9—H9A	0.9300	C11—C11^i^	1.490 (5)
C7—C6	1.383 (3)	C11—H11A	0.9700
C7—C8	1.390 (3)	C11—H11B	0.9700
C7—H7A	0.9300		
			
N2^i^—Zn1—N2	100.78 (9)	C6—C7—C8	119.04 (19)
N2^i^—Zn1—N1	96.61 (7)	C6—C7—H7A	120.5
N2—Zn1—N1	76.61 (7)	C8—C7—H7A	120.5
N2^i^—Zn1—N1^i^	76.61 (7)	C9—C8—C7	119.1 (2)
N2—Zn1—N1^i^	96.61 (7)	C9—C8—H8A	120.4
N1—Zn1—N1^i^	169.52 (9)	C7—C8—H8A	120.4
N2^i^—Zn1—O1^i^	156.73 (6)	N2—C10—C9	122.5 (2)
N2—Zn1—O1^i^	98.79 (6)	N2—C10—H10A	118.8
N1—Zn1—O1^i^	100.06 (6)	C9—C10—H10A	118.8
N1^i^—Zn1—O1^i^	88.78 (6)	N2—C6—C7	121.72 (19)
N2^i^—Zn1—O1	98.79 (6)	N2—C6—C5	115.57 (17)
N2—Zn1—O1	156.73 (6)	C7—C6—C5	122.69 (18)
N1—Zn1—O1	88.78 (6)	C1—N1—C5	118.56 (17)
N1^i^—Zn1—O1	100.06 (6)	C1—N1—Zn1	125.22 (14)
O1^i^—Zn1—O1	65.64 (8)	C5—N1—Zn1	116.18 (13)
N2^i^—Zn1—S1	129.61 (4)	N1—C5—C4	121.68 (19)
N2—Zn1—S1	129.61 (4)	N1—C5—C6	115.19 (17)
N1—Zn1—S1	95.24 (4)	C4—C5—C6	123.12 (18)
N1^i^—Zn1—S1	95.24 (4)	C3—C4—C5	119.26 (19)
O1^i^—Zn1—S1	32.82 (4)	C3—C4—H4A	120.4
O1—Zn1—S1	32.82 (4)	C5—C4—H4A	120.4
O2—S1—O2^i^	110.95 (12)	N1—C1—C2	122.7 (2)
O2—S1—O1	110.94 (9)	N1—C1—H1A	118.7
O2^i^—S1—O1	109.50 (8)	C2—C1—H1A	118.7
O2—S1—O1^i^	109.50 (8)	C3—C2—C1	118.8 (2)
O2^i^—S1—O1^i^	110.94 (9)	C3—C2—H2A	120.6
O1—S1—O1^i^	104.85 (12)	C1—C2—H2A	120.6
O2—S1—Zn1	124.53 (6)	C2—C3—C4	119.0 (2)
O2^i^—S1—Zn1	124.53 (6)	C2—C3—H3A	120.5
O1—S1—Zn1	52.43 (6)	C4—C3—H3A	120.5
O1^i^—S1—Zn1	52.43 (6)	C11—O3—H3	109.5
C10—N2—C6	118.62 (17)	O3—C11—C11^i^	113.7 (2)
C10—N2—Zn1	125.01 (14)	O3—C11—H11A	108.8
C6—N2—Zn1	116.37 (13)	C11^i^—C11—H11A	108.8
S1—O1—Zn1	94.75 (8)	O3—C11—H11B	108.8
C8—C9—C10	119.0 (2)	C11^i^—C11—H11B	108.8
C8—C9—H9A	120.5	H11A—C11—H11B	107.7
C10—C9—H9A	120.5		
			
N2^i^—Zn1—S1—O2	−114.55 (10)	N1^i^—Zn1—O1—S1	84.03 (8)
N2—Zn1—S1—O2	65.45 (10)	O1^i^—Zn1—O1—S1	0.0
N1—Zn1—S1—O2	−11.49 (9)	C10—C9—C8—C7	−0.5 (3)
N1^i^—Zn1—S1—O2	168.51 (9)	C6—C7—C8—C9	−0.5 (3)
O1^i^—Zn1—S1—O2	88.97 (11)	C6—N2—C10—C9	0.1 (3)
O1—Zn1—S1—O2	−91.03 (11)	Zn1—N2—C10—C9	−179.13 (15)
N2^i^—Zn1—S1—O2^i^	65.45 (10)	C8—C9—C10—N2	0.8 (3)
N2—Zn1—S1—O2^i^	−114.55 (10)	C10—N2—C6—C7	−1.2 (3)
N1—Zn1—S1—O2^i^	168.51 (9)	Zn1—N2—C6—C7	178.11 (14)
N1^i^—Zn1—S1—O2^i^	−11.49 (9)	C10—N2—C6—C5	−179.68 (17)
O1^i^—Zn1—S1—O2^i^	−91.03 (11)	Zn1—N2—C6—C5	−0.4 (2)
O1—Zn1—S1—O2^i^	88.97 (11)	C8—C7—C6—N2	1.4 (3)
N2^i^—Zn1—S1—O1	−23.52 (9)	C8—C7—C6—C5	179.77 (18)
N2—Zn1—S1—O1	156.48 (9)	N2^i^—Zn1—N1—C1	80.70 (17)
N1—Zn1—S1—O1	79.54 (9)	N2—Zn1—N1—C1	−179.74 (18)
N1^i^—Zn1—S1—O1	−100.46 (9)	N1^i^—Zn1—N1—C1	129.77 (17)
O1^i^—Zn1—S1—O1	180.0	O1^i^—Zn1—N1—C1	−83.00 (17)
N2^i^—Zn1—S1—O1^i^	156.48 (9)	O1—Zn1—N1—C1	−18.01 (17)
N2—Zn1—S1—O1^i^	−23.52 (9)	S1—Zn1—N1—C1	−50.23 (17)
N1—Zn1—S1—O1^i^	−100.46 (9)	N2^i^—Zn1—N1—C5	−97.15 (14)
N1^i^—Zn1—S1—O1^i^	79.54 (9)	N2—Zn1—N1—C5	2.41 (13)
O1—Zn1—S1—O1^i^	180.0	N1^i^—Zn1—N1—C5	−48.08 (13)
N2^i^—Zn1—N2—C10	−87.46 (16)	O1^i^—Zn1—N1—C5	99.15 (14)
N1—Zn1—N2—C10	178.23 (17)	O1—Zn1—N1—C5	164.14 (14)
N1^i^—Zn1—N2—C10	−9.89 (17)	S1—Zn1—N1—C5	131.92 (13)
O1^i^—Zn1—N2—C10	79.90 (16)	C1—N1—C5—C4	−0.5 (3)
O1—Zn1—N2—C10	125.73 (18)	Zn1—N1—C5—C4	177.47 (15)
S1—Zn1—N2—C10	92.54 (16)	C1—N1—C5—C6	178.66 (17)
N2^i^—Zn1—N2—C6	93.31 (13)	Zn1—N1—C5—C6	−3.3 (2)
N1—Zn1—N2—C6	−1.00 (13)	N2—C6—C5—N1	2.5 (2)
N1^i^—Zn1—N2—C6	170.88 (13)	C7—C6—C5—N1	−176.01 (17)
O1^i^—Zn1—N2—C6	−99.33 (13)	N2—C6—C5—C4	−178.35 (18)
O1—Zn1—N2—C6	−53.5 (2)	C7—C6—C5—C4	3.2 (3)
S1—Zn1—N2—C6	−86.69 (13)	N1—C5—C4—C3	0.1 (3)
O2—S1—O1—Zn1	118.12 (8)	C6—C5—C4—C3	−179.02 (18)
O2^i^—S1—O1—Zn1	−119.09 (8)	C5—N1—C1—C2	0.8 (3)
O1^i^—S1—O1—Zn1	0.0	Zn1—N1—C1—C2	−177.02 (15)
N2^i^—Zn1—O1—S1	161.88 (7)	N1—C1—C2—C3	−0.6 (3)
N2—Zn1—O1—S1	−51.09 (18)	C1—C2—C3—C4	0.1 (3)
N1—Zn1—O1—S1	−101.62 (8)	C5—C4—C3—C2	0.1 (3)

Symmetry codes: (i) −*x*, *y*, −*z*+1/2.

### Hydrogen-bond geometry (Å, °)

**Table:**

*D*—H···*A*	*D*—H	H···*A*	*D*···*A*	*D*—H···*A*
O3—H3···O2	0.82	1.97	2.746 (2)	158
